# Supplementary material for: Web engine for tumor pathology image retrievals on massive scales
Source: bioRxiv. 2025 Oct 26:2025.10.25.684566. Preprint. [Version 1] doi: 10.1101/2025.10.25.684566 (PMC12747273; doi:10.1101/2025.10.25.684566)
Supplement: Supplement 1 [file NIHPP2025.10.25.684566v1-supplement-1.pdf]

bioRxiv preprint doi: https://doi.org/10.1101/2025.10.25.684566; this version posted October 26, 2025. The copyright holder for this preprint (which was not certified by peer review) is the author/funder. This article is a US Government work. It is not subject to copyright under 17 USC 105 and is also made available for use under a CC0 license.

Supplementary Tables

Supplementary Table 1: Catalog of tumor spatial transcriptomics profiles paired with HE images. Available as file “Supp_Table_1.xlsx”

bioRxiv preprint doi: https://doi.org/10.1101/2025.10.25.684566; this version posted October 26, 2025. The copyright holder for this preprint (which was not certified by peer review) is the author/funder. This article is a US Government work. It is not subject to copyright under 17 USC 105 and is also made available for use under a CC0 license.

	Interpretation
Cytoplasmic Translation	Deregulation of protein translation is a common feature of cancer and a target of anticancer therapy[74].
Antigen Processing & Presentation	Involved in anti-tumor immune response by T cells.
Humoral Immune Response	Anti-tumor immunoglobulin responses may antagonize cancer growth[75].
Collagen	Collagen deregulation can promote cancer progressions[76].
Adaptive Immune Response	Involved in anti-tumor immune response through the adaptive immune cycle.
External Encapsulating Structure	External encapsulating structures, such as fibrotic stromas, are common morphologies in tumors[76].
Leukocyte Mediated Cytotoxicity	Involved in anti-tumor immune response by cytotoxic cells killing tumor cells.
Leukocyte Mediated Immunity	Involved in anti-tumor immune response.
Cell Killing	Involved in anti-tumor immune response by cytotoxic cells killing tumor cells.
Complement Activation	The complement system's role in cancer is controversial and can act as either a negative or positive regulator of tumorigenesis[64].
Immune Effector Process	Involved in anti-tumor immune response.

Supplementary Table 2: Cancer functional associations of top enriched pathways.

Supplementary Table 3: Data availability summary for multimodal datasets. The tables list the maps between tumor case ID, image file names, availability of mutations, and transcriptomics, utilized in the encoder training.
Available as file “Supp_Table_3.xlsx”
